# Hip Exoskeleton for Cycling Assistance

**DOI:** 10.3390/bioengineering11070683

**Published:** 2024-07-05

**Authors:** Martin Grimmer, Guoping Zhao

**Affiliations:** 1 Institute for Sport Scienceand Department of Electrical Engineering and Information Technology, Technical University of Darmstadt, 64289 Darmstadt, Germany; martin.grimmer@tu-darmstadt.de; 2 School of Mechanical Engineering, Southeast University, Nanjing 211102, China

**Keywords:** exoskeleton, assistance, cycling, optimization, hip, wearable robotics, human-in-the-loop optimization, effort, metabolic cost

## Abstract

Cycling stands as one of the most widely embraced leisure activities and serves purposes such as exercise, rehabilitation, and commuting. This study aimed to assess the feasibility of assisting three unimpaired participants (age: 34.0 ± 7.9 years, height: 1.86 ± 0.02 m, weight: 75.7 ± 12.7 kg) using the GuroX hip exoskeleton, originally designed for walking assistance, during cycling against a resistance of 1 W/kg. The performance evaluation employed a sweep protocol that manipulated the timing of the exoskeleton’s peak extension and flexion torque in addition to human-in-the-loop optimization to enhance these timings based on metabolic cost. Our findings indicate that with a peak assistance torque of approximately 10.3 Nm for extension and flexion, the GuroX substantially reduced the net metabolic cost of cycling by 31.4 ± 8.1% and 26.4 ± 14.1% compared to transparent and without exoskeleton conditions, respectively. This demonstrates the significant potential of a hip exoskeleton developed for walking assistance to profoundly benefit cycling. Additionally, customizing the assistance strategy proves beneficial in maximizing assistance. While we attribute the average motor power to be a major contributor to the reduced cycling effort, participant feedback suggests that user comfort and synchronization between the user and exoskeleton may have played integral roles. Further research should validate our initial findings by employing a larger participant pool in real-world conditions. Incorporating a more diverse set of parameters for the human-in-the-loop optimization could enhance individualized assistance strategies.

## 1. Introduction

As individuals age, there are inevitable declines in physical strength [1,2] and endurance [3] that lead to a reduction in overall physical capacity. Additionally, various diseases affecting the circulatory, respiratory, musculoskeletal, or neurological systems can further limit physical capabilities [4]. Such limitations can significantly impact daily activities, including walking and cycling, which individually represent up to 45% and 25% of daily trips, respectively [5]. Both walking and cycling contribute significantly to independence and fostering positive health outcomes across multiple domains, including cardiorespiratory, metabolic, musculoskeletal, functional, and mental health domains, as well as cancer and fall prevention [6].

To preserve and potentially enhance personal mobility while harnessing these additional health benefits, various solutions have been proposed, such as training regimens or assistive technologies. Lower limb exoskeletons have shown promise in assisting the hip, knee, or ankle, thereby reducing the effort required for walking [7]. Similarly, technical support for cycling can be achieved through electric bicycles, which decrease the effort needed for cycling and increase average speed and enjoyment [8]. While electric bicycles with hand throttles can assist up to 100% of the human cycling effort, autonomous exoskeletons designed to aid walking have shown modest reductions in walking effort. These exoskeletons typically achieve decreases of less than 20% in the net metabolic cost of walking [7] due to the system weight, inertia, and limited individualization of the assistance strategy, that defines the amplitude and timing of assistance. Nevertheless, recent advancements such as a tethered exoskeleton supporting the hip, knee, and ankle have demonstrated the potential to reduce walking effort by up to 50% after optimizing the assistance strategy for individuals’ needs [9].

Various design and control concepts have been explored for exoskeletons to assist users in diverse terrains [10,11,12]. Many of these designs incorporate gears or mechanisms to effectively transfer motor torques to different joints [7,9,13,14,15]. Methods such as machine learning and modeling are used to accurately estimate the user movement [16,17]. Typical sensor inputs for these methods are interaction forces between the user and the exoskeleton, the user and the environment, and joint or segment kinematics [13,16,18].

Our research aimed to explore the potential of the GuroX hip exoskeleton [19] to assist with cycling despite it being originally designed to assist with walking. While previous concepts have suggested using exoskeletons during cycling for either providing assistance or as part of training regimens [20], no investigation has explored the potential for reducing cycling effort. The GuroX uses a single motor in parallel with each hip to effectively transfer torque for hip flexion and extension. While ground reaction forces are utilized to control the exoskeleton during walking, the kinematics of the crank are used for control during cycling. Cycling involves two distinct phases: (1) a pushing phase from the top dead center of the crank (TDC, crank angle 0°, 0% pedal stroke) to bottom dead center (BDC, crank angle 180°, 50% pedal stroke) and (2) a pulling phase from the BDC back to the TDC. Typically, cycling demands significant energy from major muscle groups at the hip, knee, and ankle. Research indicates that the hip contributes approximately 50% of total joint power; the knee, around 40%; and the ankle, about 10% at cadences up to 70 rpm [21]. Thus, supporting hip function through an exoskeleton could offer substantial benefits. However, designing an effective assistance strategy for supporting hip extension and flexion during cycling remains an open challenge.

In the context of aiding walking, assistance strategies are typically derived from human biomechanic quantities such as muscle activity, joint moments, and joint power patterns [22]. Such approaches could inspire cycling assistance as well. For cycling, specific muscle activity patterns have been observed. As a hip extensor, the gluteus maximus becomes active before the TDC at 83% of the pedal stroke, reaches its peak activity at 15%, and returns to lower activity levels by 31%. The multiarticular rectus femoris, which flexes the hip and extends the knee, exhibits significant activity starting at approximately 67%, peaks at the TDC (0%), and then returns to lower activity levels by 31% of the pedal stroke [23]. The hip generates extension moments starting from 93% to 8% and ending at 44% to 71% of the pedal stroke. Flexion moments encompass the remainder of the pedal stroke. Within these phases, maximum extension moments of 0.5–1.5 Nm/kg and maximum flexion moments of 0.12–0.5 Nm/kg are achieved with a maximum cycling power output from 1.7 W/kg to 5.5 W/kg [24,25]. This information about timings and amplitudes makes it feasible to design an assistance strategy for the GuroX hip exoskeleton to effectively support hip extension and flexion during cycling.

It is essential to recognize that the effectiveness of an assistance strategy to reduce cycling effort may vary significantly among individuals, which is similar to that observed when applying the same strategy to different participants during walking. For example, when assessing the impact of the Harvard exosuit on the net metabolic cost of walking, the mean reduction was approximately 23%. However, the participant who benefited the least experienced a reduction of only 15%, while the participant who benefited the most achieved a reduction of 36% [14]. Similar variations in outcomes have been documented with other exoskeletons and assistance strategies as well [15]. Thus, it is clear that it is necessary to personalize and tailor the assistance strategy to the individual. Tailoring the assistance can be performed by conducting a brute force search or using more time-efficient methods such as human-in-the-loop (HITL) optimization. For example, it has been demonstrated that tailored assistance based on HITL optimization can decrease the net metabolic cost of walking by up to 50% using metabolic cost as feedback [9].

In this study, we aimed at demonstrating that a hip exoskeleton, initially developed for walking assistance, can be effectively repurposed to substantially lower the metabolic cost of cycling as measured by the net metabolic cost. To explore a range of assistance strategies, we employed two methods: (1) a brute force search with a sweep protocol that evaluates a large range of solutions and (2) human-in-the-loop optimization. The sweep protocol linearly adjusted the timing of a 10 Nm peak extension torque ranging from 10% to 40% of the pedal stroke and a 10 Nm peak flexion torque ranging from 60% to 90% of the pedal stroke. The HITL optimization used the peak timing of extension and flexion torque as a free parameter and the net metabolic cost as the optimization criterion. HITL optimization was performed to validate the results of the sweep protocol and to explore its potential to identify an individualized assistance strategy in cycling. We hypothesized that both methods are able to identify similar solutions that reduce the net metabolic cost of cycling. While maintaining the peak torque, we assumed that the fixed kinematics of the bicycle and the predefined cycling cadence favor assistance strategies with rapid increases in the exoskeleton torque and therefore provide increased average motor power to assist the participants.

## 2. Materials and Methods

Three male participants with an average age of 34 ± 7.9 years, height of 1.86 ± 0.02 m and mass of 75.7 ± 12.7 kg participated in the study. The participants had not undergone professional cycling training and possessed low to moderate experience in cycling, primarily in commuting and recreational settings. Exoskeleton experiments were approved by the Ethics Committee of TU Darmstadt (EK 06/2024). The experiment was conducted in accordance with the principles outlined in the Declaration of Helsinki. Participants provided written informed consent.

### 2.1. Experimental Setup

**Figure 1:**
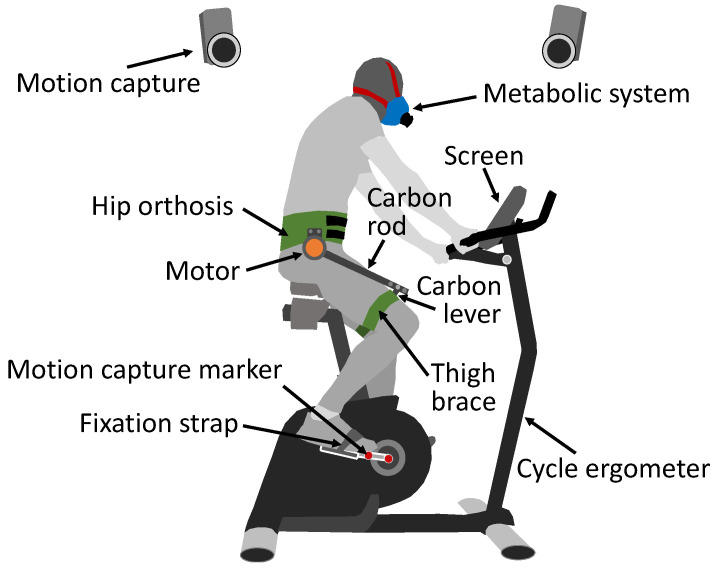
Experimental setup including cycle ergometer with screen, motion capture with two markers at each crank (red), the participant wearing the metabolic system (blue), and the GuroX hip exoskeleton. The exoskeleton includes the human–machine interface consisting of the hip orthosis and both thigh braces (green) and one motor (orange) at each hip, which use a carbon rod and a carbon lever to apply the motor torque to each thigh.

**Figure 2:**
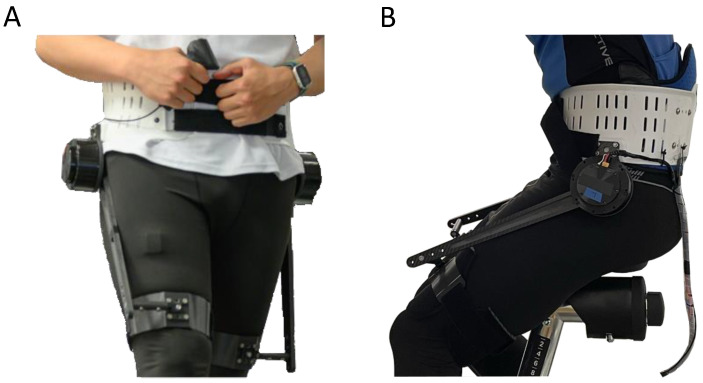
GuroX hip exoskeleton developed for lower limb assistance, perturbations, and training shown for scenarios of assisted (**A**) walking and (**B**) cycling.

A cycle ergometer (Cycle 4000, Ergofit, Germany) was used to enable cycling within the lab (Figure 1). The pedals were designed to secure the participant’s shoes using a strap, enabling both pushing and pulling motions. The metabolic cost was recorded by means of indirect calorimetry using a breath-by-breath approach (K5, Cosmed, Italy). The kinematics of both cranks were recorded at 100 Hz using motion capture(Oqus, Qualisys, Sweden) by affixing two reflective markers on each crank. The hip exoskeleton GuroX (Figure 2) was used to provide extension and flexion torques at each hip joint using one motor in parallel with each hip joint. Exoskeleton data were recorded at 1 kHz.

### 2.2. Exoskeleton Hardware and Control

The hip exoskeleton GuroX consists of two onboard quasi-direct drive BLDC motors (AK10-9, CubeMars, Nanchang, China) to generate flexion and extension torques at each hip [19]. The motor has an integrated 12-bit encoder and an planetary gearbox with a gear ratio of 9:1. The rated output torque was 18 Nm. The applied hip torque was calculated using the motor current, torque constant, and the gear ratio because of the low transmission ratio and the high backdrivability [26,27]. The exoskeleton was controlled in real time using an off-board computer with Matlab Simulink xPC (Matlab R2020a, Mathworks, Natick, MA, USA) at 1 kHz. The exoskeleton controller computes the crank angle based on the marker positions sent from the motion capture system at 100 Hz in real time via Universal Datagram Protocol (UDP). To ensure smooth desired torque patterns, the crank angle data were upsampled to a 1 kHz data rate and filtered by a second-order Butterworth low-pass filter with a cutoff frequency of 20 Hz. The mass of the exoskeleton is 3.1 kg.

**Figure 3:**
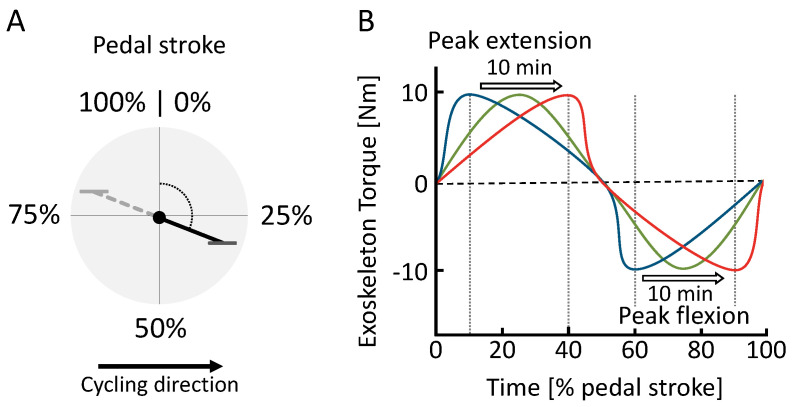
(**A**) Definition of the pedal stroke timing based on ϕ from 0% to 100% and (**B**) hip exoskeleton torque τ during the sweep session. Within the pedal stroke, peak extension ($\phi_{ext}^{p}$) and peak flexion ($\phi_{fle}^{p}$) torque timing change over the course of 15 min from early (blue) to late (red).

We parameterized the exoskeleton hip torque pattern as a function of the crank angle with one control parameter $\phi_{ext}^{p}$ (Figure 3). The torque pattern is defined as

(1) $$\tau =\left\{\begin{array}{cc} \frac{A}{2}\left(-\cos\pi \frac{\phi }{\phi_{ext}^{p}}+1\right) & 0<\phi \leq \phi_{ext}^{p}\\ \frac{A}{2}\left(\cos\pi \frac{\phi -\phi_{ext}^{p}}{0.5-\phi_{ext}^{p}}+1\right) & \phi_{ext}^{p}<\phi \leq 0.5\\ \frac{A}{2}\left(\cos\pi \frac{\phi -0.5}{\phi_{fle}^{p}-0.5}-1\right) & 0.5<\phi \leq \phi_{fle}^{p}\\ \frac{A}{2}\left(-\cos\pi \frac{\phi -\phi_{fle}^{p}}{1-\phi_{fle}^{p}}-1\right) & \phi_{fle}^{p}<\phi \leq 1 \end{array}\right.$$

where *A* is a constant denoting the maximum hip torque, which was set to 10 Nm for both hip flexion and extension, ϕ=θ/2π is the normalized crank angle (pedal stroke in percentage), and θ is the crank angle. An angle of zero is defined as the position where the pedal is at the highest point (TDC). $\phi_{ext}^{p}$ and $\phi_{fle}^{p}$ denote the normalized angle of the peak extension torque and the peak flexion torque, respectively. Considering the extension motion and the flexion motion are symmetric during cycling, we defined $\phi_{fle}^{p}=\phi_{ext}^{p}+0.5$.

The exoskeleton was fit to the participant using a modified hip orthosis made from polypropylene and a thigh brace at each thigh made from carbon fiber. All interfaces were cushioned on the inside. A carbon fiber rod on the outside of each thigh is used to transfer the torque to the thigh. Exoskeleton torque is transmitted through the end of each carbon fiber rod to the middle of each thigh brace via a rigid connecting lever.

### 2.3. Experimental Protocol

**Figure 4:**
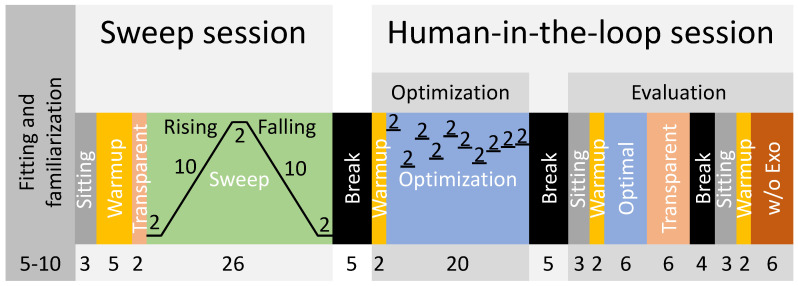
Experimental protocol including the fitting and familiarization and two experimental sessions: the sweep and the human-in-the-loop sessions. The human-in-the-loop session consists of the optimization and the evaluation part. The numbers indicate condition times in minutes.

After fitting the spiroergometry system, the hip exoskeleton, and the seat position of the bicycle ergometer to the participants preference, participants began cycling at the nearest possible resistance to 1 W/kg based on body mass (adjustable by 5 W intervals) to become familiar with cycling with the hip exoskeleton without assistance (Figure 4). Participants were instructed to monitor their cadence on the screen and adjust it to maintain a cadence between 65 and 70 rpm. This is below the preferred 80 to 90 rpm found for young adults with no to moderate cycling experience [28]. However, increasing factors such as age, cycling duration, power output or gradient will decrease preferred frequencies [29]. About 55 to 60 rpm were used for cycling training in patients with chronic stroke [30]. The resistance and the pedaling frequency were maintained throughout the entire experiment. To become acquainted with exoskeleton assistance, the exoskeleton was then activated, applying torque profiles that included peaks of 10 Nm for both hip extension and flexion. Participants continued cycling for approximately five to ten minutes until they felt comfortable with the setup.

Following this, the sweep session began with a reference sitting condition lasting three minutes. After completing a five-minute cycling warmup in transparent mode (zero motor current), a two-minute interval followed where data from cycling in the transparent mode were gathered for analysis. Subsequently, the sweep [31] was executed, comprising several stages: (1) a constant phase lasting two minutes with the earliest peak torque timing, (2) a rising phase lasting ten minutes with increasing timing, (3) another constant phase lasting two minutes with the latest timing, (4) a falling phase lasting ten minutes with decreasing timing, and (5) a final constant phase lasting two minutes with the earliest timing. During the rising phase, the timing of the peak extension torque was adjusted from 10% to 40% of the pedal stroke, and for the peak flexion torque, it was adjusted from 60% to 90%. We notably standardized the peak flexion torque to consistently maintain a 50% offset relative to the peak extension torque. This adjustment accommodates the inherent offset in muscle activity between flexors and extensors, thereby effectively minimizing the number of control parameters for both the sweep and HITL sessions. The timing adjustments during the sweep were made with a step size of 0.05% per second. After completing the sweep, participants ceased cycling and had a break of approximately five minutes to rest.

Subsequently, the human-in-the-loop session commenced with a two-minute warmup in transparent mode, followed by an optimization phase lasting 20 min. The HITL optimization was performed using a Bayesian optimization-based method as described in [32]. Bayesian optimization is a noise-tolerant global optimization method that has been successfully implemented in various HITL studies [32,33,34]. To avoid biased sampling and early convergence to a local minimal, four initial evaluation points (peak torque timings, $\phi_{ext}^{p}$) were randomly chosen within the variable bounds (10% to 40%). Six iterations were then used for finding the optimal value [32]. In total, there were ten iterations in the optimization process (Figure 4). Each iteration took 2 min, as the instantaneous metabolic rate was estimated using 2 min of transient metabolic data [35]. The *bayesopt* function, which is a function for Bayesian optimization implemented by Matlab, was used in this study. Following the optimization, participants had another break of about five minutes to rest. The second part of the human-in-the-loop session began with three minutes of sitting followed by a two-minute warmup in transparent mode. This was followed by a six-minute evaluation session to assess the identified optimal peak torque timing from the optimization phase (referred to as “Optimal”). Subsequently, six minutes in transparent mode were completed before a rest period of approximately four minutes, during which the exoskeleton was removed and the data stored. Lastly, another cycle commenced with three minutes of sitting, two minutes of warmup in transparent mode, and six minutes of cycling without the exoskeleton (w/o Exo).

Sweep, optimization, and evaluation times were selected based on the experience and outcomes of previous studies, though a primary factor was the delay of up to two minutes for changes in movement effort to be reflected in measured metabolic cost [31,35].

### 2.4. Data Analysis

#### 2.4.1. Metabolic Cost

Oxygen consumption and carbon dioxide production were used to calculate the metabolic power using a modified Brockway equation [36]. The metabolic power was normalized by the mass of each participant. To match the exoskeleton data and to eliminate irregular data due to the breath-by-breath measurement, metabolic power was interpolated to 1000 Hz. The net metabolic cost was obtained by subtracting the average metabolic power for the last two minutes of the sitting condition from all cycling conditions for each session and part separately. The relative changes in net metabolic power between the assisted and unassisted conditions were calculated similar to [37]. For the sweep session, the average of the 2 min of data from the preceding transparent condition were used for comparison. For the human-in-the-loop session, the average of the last 3 min of each transparent mode, the Optimal, and the without-exoskeleton condition were used. The average of the sweep was determined as the average of the ten-minute rising sweep and the reverse of the ten-minute falling sweep without further data adjustments [31]. To determine the minima of the net metabolic cost and its timing during the sweep session, the 1000 Hz metabolic data were filtered with a zero-lag, second-order Butterworth filter with a cut-off frequency of 0.007 Hz.

#### 2.4.2. Crank Angle and Angular Velocity

The crank angle was calculated in real time using the 3D coordinates of the two markers placed on each crank where the zero angle was defined as the TDC. The pedal stroke ranged from 0° to 360°, and pedal strokes were extracted using the transition of the crank angle to the first value equal to or larger than 0°. The crank angle data were filtered with a zero-lag, second-order Butterworth filter with a cut-off frequency of 100 Hz. The respective crank angular velocity was determined by differentiation. Crank kinematics were analyzed for the right side.

#### 2.4.3. Motor Torque and Power

The motor torque was not directly measured by a torque sensor, but rather, it was calculated using motor current, torque constant, and gear ratio. A motor encoder provided the position, which was differentiated to obtain the motor angular velocity. The motor torque was filtered with a zero-lag, second-order Butterworth filter with a cut-off frequency of 20 Hz. The exoskeleton power was determined by multiplying the motor torque and the motor angular velocity of each limb separately. The average motor power for each pedal stroke was determined as the mean of the motor power during each pedal stroke. For a comparison of the motor and crank behavior during the sweep protocol, five equally distributed phases (P1 to P5) were defined. Each phase represents the change in peak torque timing of 3% of the pedal stroke, which lasts one minute (peak extension torque P1 10–13%, P2 16.75–19.75%, P3 23.5–26.5%, P4 30.25–33.25%, P5 37–40%). The peak flexion torque timing has a fixed offset of 50% of the pedal stroke with respect to the peak extension torque timing. For all pedal strokes in these phases, participant averages and group means were calculated. Exoskeleton biomechanics were analyzed for only the right side.

#### 2.4.4. Polynomial Fit

A first-degree polynomial fit was performed with the average motor power of each pedal stroke as the independent variable and the net metabolic cost as the dependent variable. To ensure that the number of samples for each participant was equal, the net metabolic cost and the corresponding average motor power were interpolated at 1 Hz, resulting in 600 samples for each of the 10 min rising and falling parts of the sweep session. The rising and the falling part (inverse timing) were averaged. The fit was performed for each of the participants and for all participants combined (Average).

## 3. Results

### 3.1. Crank Angle and Angular Velocity

**Figure 5:**
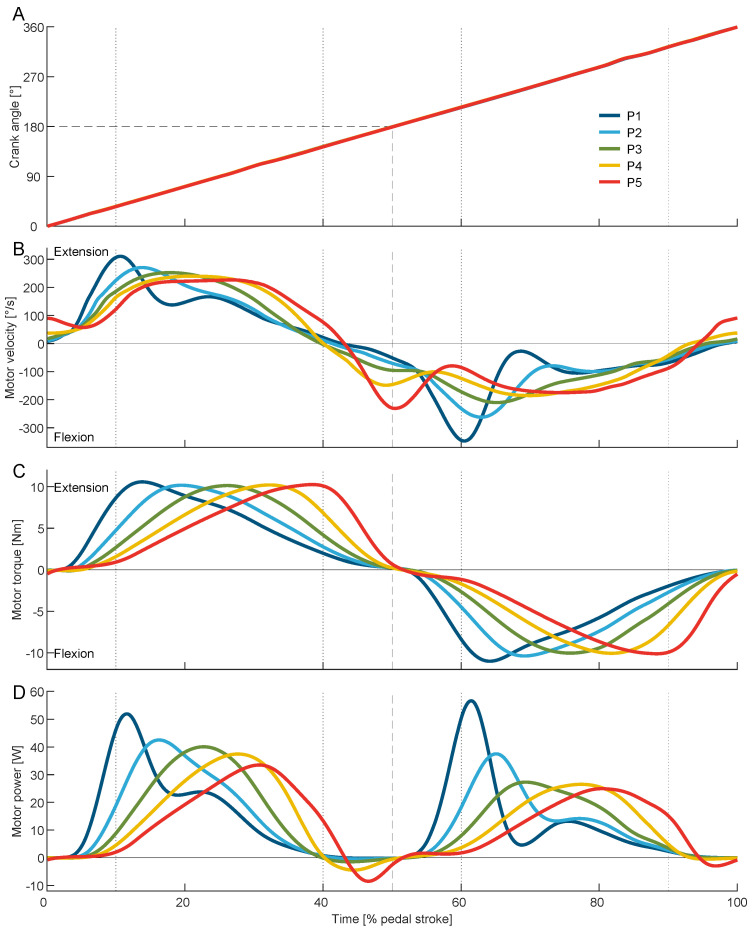
Average crank angle (**A**), motor velocity (**B**), motor torque (**C**), and motor power (**D**) during the pedal stroke for the five phases P1 to P5 (peak extension torque P1 10–13%, P2 16.75–19.75%, P3 23.5–26.5%, P4 30.25–33.25%, P5 37–40%, and peak flexion torque with 50% offset). Dotted vertical lines indicate the earliest and latest applied peak extension torque. Dashed lines indicate the middle of the time of the pedal stroke as well as the crank angle at bottom dead center (BDC). For the crank angle, the results of P1 to P5 overlap each other.

During cycling, the crank angle exhibited a consistent linear increase throughout both exoskeleton-assisted and unassisted conditions (Figure 5A). Although the torque varied throughout the sweep session, no alterations due to the exoskeleton torque were observed during phases P1 through P5. The average crank angular velocity was similar between conditions, ranging from 409 ± 13 °/s (68.2 rpm) without the exoskeleton to 424 °/s (70.7 rpm) in the Optimal and in P3 of the sweep session (Table 1).

### 3.2. Motor Torque and Power

**Figure 6:**
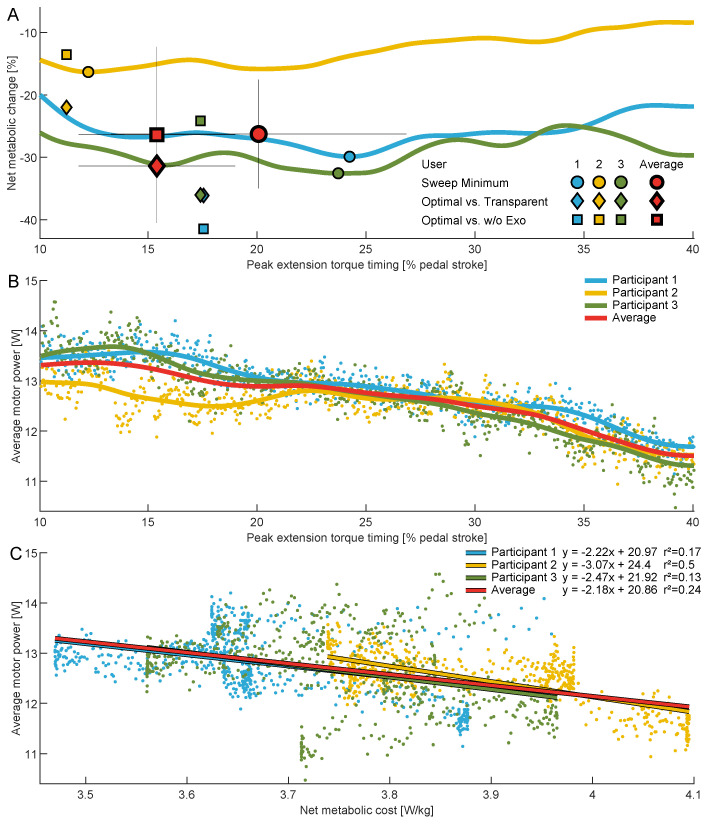
(**A**) Net metabolic change across the range of assessed torque timings during cycling relative to $\phi_{ext}^{p}$. Solid lines are participant-specific averages across the sweep session. Circles indicate sweep session minima compared to the transparent condition, diamonds indicate the optima from the human-in-the-loop optimization compared to the transparent condition, and squares indicate the optima from the human-in-the-loop optimization compared to the without exoskeleton (w/o Exo) condition.The results for each participant are shown in blue, yellow, and green, respectively. Group averages are indicated in red and include standard deviations (thin black lines). (**B**) Average motor power for each participant and the group average across the range of assessed torque timings. Data points indicate participant-specific samples used to determine the mean. (**C**) Average motor power versus net metabolic cost for the individual participants and the group average. Data points indicate participant-specific samples, and the solid lines represent first-degree polynomial fits, where coefficients and r-squared values are also provided.

To apply the desired torque, P1 used the greatest motor velocity and acceleration, while P3 used the smoothest motor velocity trajectory over the pedal stroke (Figure 5B). The exoskeleton torque changed as desired for the sweep session (Figure 5C). The peak motor torque varied slightly for the sweep session from 10.2 ± 0.1 Nm during P3 to 10.6 ± 0.2 Nm during P1. The Optimal was found to utilize a peak toque of 10.4 ± 0.3 Nm. Similar to the motor torque, the exerted motor power changed over the sweep session (Figure 5D). The average motor power decreases from 13.1 ± 0.4 W during P1 to 11.5 ± 0.3 W during P5 and was found to be greatest at 13.9 ± 1.1 W for the Optimal (Table 1, Figure 6B).

### 3.3. Metabolic Cost

At an average resistance of 0.99 ± 0.02 W/kg (75 ± 13 W), the net metabolic cost was reduced from 4.9 ± 0.4 W/kg during the transparent condition to a minimum of 3.6 ± 0.1 W/kg when being assisted during the sweep session. This is a reduction of 26.3 ± 8.7% (Table 2, Figure 6A). For the HITL session, the transparent condition required 4.9 ± 0.5 W/kg on average; the without-exoskeleton condition, 4.6 ± 0.2 W/kg; and Optimal, 3.4 ± 0.5 W/kg. The optimal condition therefore provided net metabolic cost reductions of 31.4 ± 8.1% compared to the transparent condition and 26.4 ± 14.1% compared to without the exoskeleton (Table 2, Figure 6A).

The average timings for the maximum reduction in metabolic cost for the sweep and the HITL sessions were found to be 20.1 ± 6.8% and 15.3 ± 3.6% of the pedal stroke, respectively (Table 2, Figure 6A).

### 3.4. Metabolic Cost vs. Motor Power

For the group average, we observed weak explanatory power (r^2^ range: 0.2–0.399) between the average motor power and the net metabolic cost. Individual participant data indicate that participant 2 exhibited moderate explanatory power (r^2^ range: 0.4–0.599), whereas participants 1 and 3 demonstrated very weak explanatory power (r^2^ range: 0.0–0.199) (see Figure 6C).

## 4. Discussion

By applying an average of approximately 10 Nm of peak hip extension and flexion torque with the GuroX hip exoskeleton, we were able to assist cycling with up to 13.9 W per hip joint. This provided up to 37% of the power necessary to counteract the average cycling resistance of 75.7 W (approximately 1 W/kg). As hypothesized, this resulted in notable reductions in the net metabolic cost of cycling by up to 31% compared to the transparent condition and by 26.4% compared to without the exoskeleton. To put this in perspective, a 2020 review conducted by Sawicki et al. [7] emphasized that the most significant benefits observed in aided walking with autonomous exoskeletons had reached approximately 20% [38]. This demonstrates the potential of lower limb exoskeletons to extend their assistance to various other movement activities, such as cycling.

We did not anticipate such a substantial discrepancy in the reduction in net metabolic cost between the transparent and the without-exoskeleton conditions considering that the participants were not required to carry the entire mass. We suspect that part of this difference may have been due to the resistance of the exoskeleton in the transparent mode, although the inherent noise in the metabolic cost data and the relatively small sample size in this initial assessment could have also contributed. We noticed a similar difference for the Optimal compared to the sweep minimum condition and assume, in addition to the previously-mentioned reasons, that this was due to the time delay of about two minutes that is necessary for the metabolic cost to reflect actual effort [31].

We observed comparable timings for the exoskeleton peak torque between participants 1 and 3 during both the sweep and HITL sessions, although the sweep session exhibited its optimum 7% later. However, for participant 2, the peak torque timing was 6% to 12% earlier, aligning consistently between the HITL and sweep sessions. The discrepancy in timing for participants 1 and 3 could possibly be attributed to metabolic cost signal noise and the small changes observed for the metabolic cost over a wide range of peak extension torque timings. In contrast, participant 2 displayed a distinctive trend such that there was a discernible minimum for very early peak extension torque timings, where net metabolic cost steadily increased for later timings. This monotonically increasing metabolic response could have facilitated the identification of the Optimal timing. The discovery of varying optimal timings underscores a crucial point: akin to assisted walking, cycling can also benefit from personalized assistance strategies. Moreover, employing HITL optimization based on metabolic cost should be viewed as a viable method to tailoring cycling assistance using a hip exoskeleton to individual needs.

Prior research has demonstrated that cycling requires maximum extension moments that range between 0.5 Nm/kg and 1.5 Nm/kg and maximum flexion moments that range between 0.12 Nm/kg and 0.5 Nm/kg [24,25]. In this study, we applied extension and flexion moments of about 0.13 Nm/kg. This is about equal to the flexion moment though it is approximately one-fourth of the extension moment that is found in recreational cycling. Despite the GuroX motors allowing for torque of up to 50 Nm, we chose to cap the torques at 10 Nm to ensure a high comfort level for the participants. We anticipate that augmenting the peak torques could potentially lead to a further decrease in the net metabolic cost of cycling. However, according to the feedback received from the participants, rapid increases in torque, as experienced with the early peak torques in P1, could cause discomfort especially at the anterior pelvis (spina iliaca anterior superior). Therefore, future research should also focus on developing concepts that enhance the wearing comfort of human–machine interfaces in order to enable the application of high torques.

Increased motor torques should also allow for an increased average motor power, which, as hypothesized, could be one major source for determining the level of assistance provided by the exoskeleton. A weak relationship was found between average motor power and average net metabolic cost. However, as the slope of the polynomial fit was found to be very similar for all three participants, we still believe that motor power is a strong contributor to the assistance level. In addition, the average motor power for the Optimal was about 1 W higher than in the condition with minimal metabolic cost from the sweep session. Given that the exoskeleton control remained unchanged, we infer that this improvement stemmed from participant adaptation. Preceding the identification of the Optimal, participants engaged in 46 min of cycling with exoskeleton assistance, likely facilitating familiarity and enhancing their interaction with the exoskeleton. This familiarity potentially improved the synchronization between motor and limb velocities. Additionally, the extended trial duration of six minutes with consistent assistance might have further contributed to this enhanced synchronization.

We were surprised by how adeptly all participants managed to integrate the different exoskeleton’s perturbations from P1 to P5 without compromising the almost perfect linear increase in the crank angle during the pedal stroke. However, regulating the muscles to achieve such a linear increase in crank angle might necessitate additional effort, particularly in scenarios involving highly dynamic increases in assistance torque with early peak timing such as in P1. This observation could support the preference of participants 1 and 3 for later timings with smoother motor velocity trajectories, despite providing less average motor power, compared to participant 2. Moreover, participants 1 and 3 might prefer later timings compared to participant 2 because the energy provided by the exoskeleton for hip rotation can potentially be more efficiently transferred to crank rotation. Modeling techniques could be employed to investigate these details and their implications.

## 5. Conclusions

Our main contribution is the demonstration of the feasibility and effectiveness of using a hip exoskeleton, originally designed for walking, to significantly reduce the metabolic cost of cycling. Despite peak torques of approximately 10 Nm, a capability readily attainable with most lower limb exoskeletons, we effectively mitigated the net metabolic cost of cycling. Our findings indicate that a significant reduction in the net metabolic cost stems from the average motor power provided. However, our preliminary results and participant feedback suggest that user exertion could be influenced by factors such as comfort as the exoskeleton applies torque and the effort required to synchronize the human–machine interaction. Future research should validate our initial findings by involving a larger and more diverse group of participants and testing the exoskeleton under dynamic, real-world cycling conditions. Moreover, investigations into exoskeleton-supported cycling could explore assisting other or additional joints and to incorporate a greater variety of free parameters for the human-in-the-loop optimization, thereby potentially tailoring assistance more individually. Additionally, analyzing changes in muscle activity could unveil the specific contributors to the decreased effort during cycling.

## Figures and Tables

**Table 1:** Group means (±standard deviation) of the average crank angular velocity, maximum motor torque, and average motor power for the analyzed conditions of the sweep (peak extension torque P1 10–13%, P2 16.75–19.75%, P3 23.5–26.5%, P4 30.25–33.25%, P5 37–40%) and the human-in-the-loop (HITL) sessions.

	Sweep Session						HITL Session		
	Transp.	P1	P2	P3	P4	P5	Optimal	Transp.	w/o Exo
Ave. crank ang. vel. [°/s]	420 ± 10	415 ± 11	418 ± 11	424 ± 20	423 ± 21	420 ± 31	424 ± 13	420 ± 6	409 ± 13
Max. motor torque [Nm]	-	10.6 ± 0.2	10.3 ± 0.2	10.2 ± 0.1	10.3 ± 0.1	10.3 ± 0.0	10.4 ± 0.3	-	-
Ave. motor power [W]	-	13.1 ± 0.4	13 ± 0.3	12.8 ± 0.1	12.4 ± 0.2	11.5 ± 0.3	13.9 ± 1.1	-	-

**Table 2:** Participant data and group means (±standard deviation) for the net metabolic cost (in W/kg), related peak extension torque timings (in % of pedal stroke, PS), and net metabolic reductions between conditions (in %) for the sweep and the human-in-the-loop session.

User	Sweep Session				Human-in-the-Loop Session					
	Transp.	Minimum	Timing	Transp.	Transp.	Optimal	Timing	w/o	Transp.	w/o Exo
	[W/kg]	[W/kg]	Minimum	vs.	[W/kg]	[W/kg]	Optimal	Exo	vs.	vs.
			[%PS]	Minimum			[%PS]	[W/kg]	Optimal	Optimal
				[%]					[%]	[%]
1	4.9 ± 0.4	3.5	24.3	−29.9	4.4 ± 0.6	2.8 ± 0.4	17.5	4.8 ± 0.7	−36.1	−41.5
2	4.5 ± 0.6	3.7	12.3	−16.3	4.9 ± 0.5	3.8 ± 0.8	11.2	4.4 ± 0.7	−22.0	−13.5
3	5.3 ± 0.7	3.6	23.7	−32.6	5.4 ± 0.6	3.5 ± 0.6	17.3	4.6 ± 0.8	−36.0	−24.2
Average	4.9 ± 0.4	3.6 ± 0.1	20.1 ± 6.8	−26.3 ± 8.7	4.9 ± 0.5	3.4 ± 0.5	15.3 ± 3.6	4.6 ± 0.2	−31.4 ± 8.1	−26.4 ± 14.1

## Data Availability

The raw data supporting the conclusions of this article will be made available by the authors upon reasonable request.

## Abbreviations

The following abbreviations are used in this manuscript:

BDC	bottom dead center of the crank;
TDC	top dead center of the crank;
HITL	human-in-the-loop;
Optimal	optimal condition with control parameters from HITL optimization;
PS	pedal stroke;
P1 to P5	phases during sweep protocol;
Transp.	transparent condition with zero motor current;
w/o Exo	condition without wearing the exoskeleton;
ϕ	normalized crank angle;
$\phi_{ext}^{p}$	peak extension torque timing;
$\phi_{fle}^{p}$	peak flexion torque timing;
τ	exoskeleton hip torque.
